# Beryllium Lymphocyte Proliferation Test: Differential Diagnosis of Sarcoidosis and Chronic Beryllium Disease

**DOI:** 10.1016/j.chest.2025.06.034

**Published:** 2025-07-01

**Authors:** Louis Jouanjan, Charlott Terschluse, Gernot Zissel, Prerana Agarwal, Emil Wachenfeld, Caroline Quartucci, Daniel Soriano, Joachim Müller-Quernheim, Daiana Stolz, Björn C. Frye

**Affiliations:** a Departments of Pneumology, Medical Center - University of Freiburg, Faculty of Medicine, University of Freiburg, Freiburg, Germany; b Department of Diagnostic and Interventional Radiology, Medical Center - University of Freiburg, Faculty of Medicine, University of Freiburg, Freiburg, Germany; c Institute for Occupational Health and Product Safety, Bavarian Health and Food Safety Authority, Environmental Health, Munich, Germany; d Institute and Clinic for Occupational, Social and Environmental Medicine, University Hospital, LMU Munich, Munich, Germany

**Keywords:** chronic beryllium disease, lymphocyte proliferation test, sarcoidosis

## Abstract

**Background:**

Chronic beryllium disease (CBD) is considered a phenocopy of sarcoidosis, generally caused by occupational exposure to beryllium. Its diagnosis relies on the demonstration of beryllium sensitization by the beryllium lymphocyte proliferation test (BeLPT).

**Research Question:**

How well does the BeLPT discriminate between CBD and sarcoidosis in patients with suspected beryllium exposure and what are the clinical characteristics of these 2 groups?

**Study Design and Methods:**

BeLPT results and clinical characteristics of patients with suspected beryllium exposure were retrospectively analyzed.

**Results:**

A total of 1,234 BeLPTs from 431 patients were included. Of 210 patients with established granulomatous disease, 87 patients (41.4%) were diagnosed with CBD and 106 (50.5%) were diagnosed with sarcoidosis; in 17 cases (8.1%), the BeLPT results were inconclusive. The remaining 221 patients had other diagnoses or could not be classified. A single BeLPT had a sensitivity of 61.5% (95% CI, 55.8-67.0) and a specificity of 90.8% (95% CI, 86.5-93.9). For split-sample tests, the sensitivity and specificity were 76.0% (95% CI, 67.7-82.8) and 80.4% (95% CI, 71.4-87.1), respectively. Patients with CBD had lower lung function than those with sarcoidosis, as measured by FVC (mean *z* score, −1.58 vs −0.75, respectively; *P* = .002). A restrictive pattern was more common in CBD than in sarcoidosis (41.0% vs 14.8%, respectively; OR, 3.99; *P* = .002).

**Interpretation:**

Despite low sensitivity, the BeLPT was able to detect CBD in 41.4% of patients with a granulomatous disease and suspected beryllium exposure. CBD was associated with lower lung function compared with sarcoidosis.

**Take-Home Points**

**Study Question:** Can the beryllium lymphocyte proliferation test (BeLPT) distinguish chronic beryllium disease (CBD) from sarcoidosis, and how do both diseases compare clinically?

**Results:** Despite low sensitivity, the BeLPT was shown to be able to identify CBD with high specificity; CBD was associated with lower lung function compared with sarcoidosis.

**Interpretation:** Our results suggest that in patients with apparent sarcoidosis and possible beryllium exposure, the BeLPT should be performed to exclude CBD; negative and borderline tests should be repeated.

Chronic beryllium disease (CBD) is a granulomatous disease considered clinically identical to sarcoidosis and caused by exposure to beryllium,^1^ a lightweight yet resistant metal, essential to the aerospace, nuclear, and defense industries.^2^ Exposure can lead to beryllium sensitization (BeS), notably in genetically susceptible individuals,^3^^,^^4^ some of which develop CBD.^1^

Sarcoidosis and CBD are both driven by T-helper-1-cell and macrophage activation, leading to the formation of nonnecrotizing granulomas.^5^^,^^6^ They share radiologic appearances and clinical manifestations including cough, dyspnea, and fatigue. Although sarcoidosis frequently displays extrapulmonary manifestations, these are anecdotal in CBD.^7^

The diagnosis of CBD requires evidence of granulomas on biopsy and immunologic evidence of BeS, determined by the beryllium lymphocyte proliferation test (BeLPT).^8^ Sarcoidosis is a diagnosis of exclusion for which other causes of granulomas must be ruled out.^9^ The BeLPT quantifies lymphocyte proliferation after ex vivo cultivation and stimulation with beryllium.^10^^,^^11^ It is performed using peripheral blood mononuclear cells (PBMCs) or bronchoalveolar lavage (BAL) cells.^12^^,^^13^ The BeLPT has been developed, has been studied, and is used in the United States as a surveillance tool to detect BeS and prevent development of CBD in asymptomatic beryllium-exposed workers.^10^^,^^14^, ^15^, ^16^ Outside the United States, it is generally used post hoc to rule out CBD as a differential diagnosis of sarcoidosis in patients diagnosed with granulomatous disease who might have been exposed to beryllium (e-Appendix 1).^17^^,^^18^

Our main objective was to evaluate the accuracy of the BeLPT in distinguishing between CBD and sarcoidosis in a clinical setting. The secondary objective was to characterize patients with CBD and sarcoidosis, highlighting similarities and differences in clinical presentation.

## Study Design and Methods

**Figure 1:**
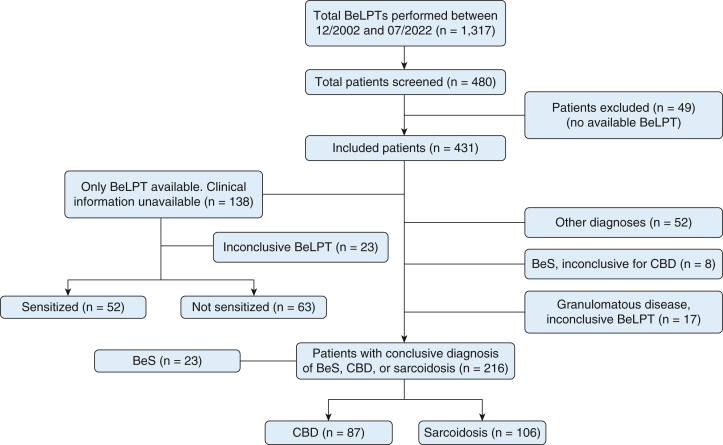
Flowchart of patient inclusion and classification. BeLPT = beryllium lymphocyte proliferation test; BeS = beryllium sensitization; CBD = chronic beryllium disease.

We performed a retrospective study of all patients who received a BeLPT at our institution between 2002 and 2022. Patients were included if at least 1 documented BeLPT was performed during the study period. Exclusion criterion was the absence of an available BeLPT report (Fig 1). We retrospectively analyzed data from each patient’s first presentation and first follow-up, including general characteristics, patient history, and key clinical parameters such as pulmonary function test (PFT) results, serum levels for neopterin, soluble interleukin 2 receptor, and angiotensin-converting enzyme, BAL cell counts, and biopsy results.

All procedures were performed in accordance with the Helsinki Declaration of 1964 and the ethical standards of our institutional ethics committee. Approval was obtained from the institutional ethics committee of the Medical Faculty of the University of Freiburg (approval No. 24-1284-S1-retro). All data were analyzed after pseudonymization.

### Beryllium Lymphocyte Proliferation Test

Mononuclear cells from blood or BAL were cultured with either beryllium sulphate in 6 concentrations ranging from 100 pM to 10 μM, with phytohemagglutinin A and concanavalin A as positive controls, or without stimulation as negative control. We avoid higher beryllium sulphate concentrations of 100 μM because they result in cell toxicity in our patients (in contrast with healthy screening populations). Lymphocyte proliferation was measured by enzyme-linked immunosorbent assay on days 4 and 7, after incorporation of 5-bromo-2'-deoxyuridine on days 3 and 6 as previously described.^19^ To avoid radiation, we use the 5-bromo-2'-deoxyuridine method, which follows the same principles as the tritiated thymidine method and has been shown to yield equivalent results.^19^

The BeLPT was performed before initiation, after discontinuation, or at the lowest achievable level of immunosuppressive therapy. For the BeLPT from PBMCs, we set up 2 parallel assays from each sample (split-sample testing).

The BeLPT was classified as negative when the mean optical density of each beryllium concentration at days 4 and 7 was below the negative control mean optical density + 2 SDs, as borderline if at least 1 concentration lay > 2 SDs, but < 2 concentrations were > 3 SDs, and as positive if at least 2 concentrations were > 3 SDs (e-Appendix 2).

### Diagnostic Classification

The diagnostic criteria of the American Thoracic Society (ATS) were used as reference standard for the diagnosis of BeS and CBD.^8^ BeS was defined as follows: at least 2 positive BeLPT results, 1 positive and 1 borderline BeLPT, or 1 positive BAL BeLPT. For CBD, patients had to fulfill BeS criteria and display granulomas or findings which would otherwise have been diagnosed as sarcoidosis. Sarcoidosis was diagnosed when ATS diagnostic criteria for sarcoidosis were met and BeS was ruled out by the BeLPT.^9^ In all cases, other causes of granuloma were excluded during routine workup (e-Appendix 1). The diagnoses of CBD and sarcoidosis established by the treating physicians were reviewed and considered conclusive in the presence of biopsy-proven granulomas and a compatible clinical presentation. Cases without available biopsy were considered conclusive only with highly suggestive clinical and radiologic manifestation or if previously established elsewhere.

PFTs were performed on a Jaeger MasterScreen (Jaeger). FVC, FEV_1_, FEV_1_/FVC ratio, total lung capacity, and diffusion capacity for carbon monoxide (Dlco) were reported as *z* scores. PFT patterns were classified as restrictive, obstructive, mixed, isolated diffusion impairment, or normal according to current European Respiratory Society/ATS standards.^20^

### Statistical Analysis

Statistical analyses were conducted using Prism version 9.5.1 (GraphPad Software Inc). Figures were created with Prism version 9.5.1 and R version 4.2.3 (R Core Team), using the eulerr package version 7.0.2 (Johan Larsson). Continuous variables were reported as mean and SD or median and interquartile range; categorical variables were reported as absolute numbers and percentages. Percentages were based on available data only. Categorical variables were compared using Fisher exact test, and effect sizes were reported as OR and 95% CI. Continuous variables were compared using paired or unpaired Student or Mann-Whitney *U* test, and effect sizes were reported as mean/median difference and 95% CI. Alpha level was .05.

To calculate test accuracies, in the absence of a criterion standard, true positives were defined as patients who at the end of diagnostic evaluation met ATS sensitization criteria, and true negatives were defined as those who did not. For statistical analysis, the 3 possible test outcomes (negative, borderline, and positive) were reduced to a dichotomous outcome (negative or positive), where borderline results were either excluded, counted as negative (high-threshold approach), or counted as positive (low-threshold approach). Likelihood ratios (LRs) were calculated to further assess the predictive value of each outcome separately and independently of relative frequency.

## Results

We identified 480 patients who underwent a BeLPT, totaling 1,317 BeLPT tests. After excluding 49 patients and 83 BeLPT tests (e-Table 1, Fig 1, 431 patients and 1,234 BeLPT tests were included. Of these tests, 1,084 (87.8%) were from PBMCs and 150 (12.2%) were from BAL.

Patients were categorized as having CBD (n = 87), sarcoidosis (n = 106), or BeS without CBD (n = 23) (Fig 1). Some cases were classified as uncertain: granulomatous lung disease where classification as CBD or sarcoidosis was uncertain because of inconclusive BeLPT results (n = 17) and BeS with uncertain CBD, mostly because of missing histologic proof (n = 8). Patients who did not fit any of these categories were grouped under alternative diagnoses (n = 52). A further 138 patients without available clinical data for whom we could only state on the presence of BeS, but not on the presence of granulomatous disease, were categorized as sensitized (n = 52), not sensitized (n = 63), and inconclusive BeLPT (n = 23). Table 1 reports the baseline characteristics of the overall study population and of those classified as definite CBD or sarcoidosis. A total of 84 patients (95.5%) with CBD and 86 patients (81.1%) with sarcoidosis had known histologic evidence of granuloma (e-Table 2).

**Table 1:** Baseline Characteristics of the General Study Population and Relevant Subclasses

Characteristic	Overall (N = 431)	CBD (n = 87)	Sarcoidosis (n = 106)	*P* Value^a^
General characteristics				
Age, y	49.8 [12.9]	51.7 [12.1]	45.4 [11.9]	< .001^b^
BMI, kg/m^2^	27.3 [4.3]	28.4 [4.3]	26.9 [4.3]	.072
Sex available	405	87	106	
Female	82 (20.2)	15 (17.2)	25 (23.6)	.464
Male	323 (79.8)	72 (82.8)	81 (76.4)	.464
Smoking status				
Smoking status available	137	50	58	
Never smoked	52 (40.0)	16 (32.0)	29 (50.0)	.078
Actively smokes	24 (17.5)	11 (22.0)	7 (12.1)	.201
Formerly smoked	61 (44.5)	23 (46.0)	22 (37.9)	.438
Pack-years	12.7 [18.7]	15.1 [21.9]	8.7 [15.6]	.094
Comorbidities				
Comorbidities available	197	76	82	
COPD	12 (6.1)	4 (5.3)	3 (3.7)	.712
Asthma	16 (8.1)	9 (11.8)	6 (7.3)	.419
OSA	15 (7.6)	6 (7.9)	5 (6.1)	.759
AHT	48 (24.4)	23 (30.3)	16 (19.5)	.351
Immunosuppressive treatment				
Treatment available	204	75	84	
None	174 (85.3)	63 (84.0)	71 (84.5)	> .999
Corticosteroids	29 (14.2)	12 (16.0)	12 (14.3)	.826
Low dose^c^	22 (10.8)	12 (16.0)	9 (10.7)	.356
High dose^c^	3 (1.5)	0 (0)	1 (1.2)	> .999
Dose unknown	4 (2.0)	0 (0)	2 (2.4)	.498
Second-line treatment^d^	5 (2.5)	5 (6.7)	0 (0)	.022^b^
TNF inhibitor^e^	1 (0.5)	0 (0)	1 (1.2)	> .999
Scadding stage				
Imaging available	101	57	44	
Scadding 0	14 (13.9)	7 (12.3)	7 (15.9)	.773
Scadding 1	25 (24.8)	14 (24.6)	11 (25.0)	> .999
Scadding 2	38 (37.6)	21 (36.8)	17 (38.6)	> .999
Scadding 3	6 (5.9)	3 (5.3)	3 (6.8)	> .999
Scadding 4	18 (17.8)	12 (21.1)	6 (13.6)	.435

Data are presented as mean [SD], No. (%), No., or as otherwise indicated. Percentages are based on available data. Baseline refers to time of first beryllium lymphocyte proliferation test. Scadding stages are as follows: 0 (no hilar adenopathy, no parenchymal involvement), 1 (hilar adenopathy, no parenchymal involvement), 2 (hilar adenopathy and parenchymal involvement), 3 (no hilar adenopathy, parenchymal involvement), and 4 (fibrotic lung disease). AHT = arterial hypertension; CBD = chronic beryllium disease; TNF = tumor necrosis factor.

a *P* value refers to CBD vs sarcoidosis.

b Significant at α = .05.

c Low dose ≤ 7.5 mg prednisone; high dose > 7.5 mg prednisone.

d Azathioprine (n = 3) or methotrexate (n = 2), each in combination with low-dose corticosteroids.

e Adalimumab.

**Figure 2:**
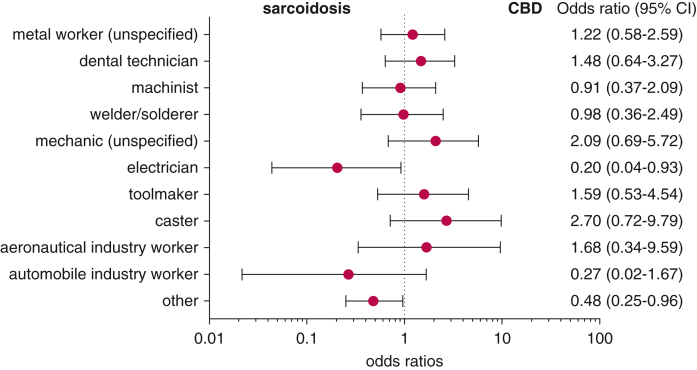
ORs for the association of a field of occupation with CBD vs sarcoidosis presented as forest plots. Precision mechanics excluded (only 2 individuals, both in the CBD group). CBD = chronic beryllium disease.

Most patients had occupational exposure to metals. The most represented occupations were unspecified metal workers and dental technicians, followed by machinists. No occupation was overrepresented in CBD; however, electricians were underrepresented in CBD (e-Table 3, Fig 2).

### BeLPT Results

Of all BeLPT tests, 375 (30.4%) were positive, 202 (16.4%) were borderline, and 613 (49.7%) were negative. A total of 44 (3.6%) were uninterpretable. Sensitized patients had an average of 0.5 and up to 5 negative tests before first testing positive (e-Table 4). Sensitized patients had an average of 3.6 tests and nonsensitized patients had 2.3 tests performed overall (e-Table 5).

### All Sensitized vs Nonsensitized

A single positive BeLPT had a sensitivity of 64.5% (95% CI, 60.2-68.6) and a specificity of 93.9% (95% CI, 91.1-95.8) (Table 2). These numbers did not significantly change when only considering patients who had at least 2 (sensitivity, 64.6%; specificity, 93.3%) or at least 3 tests (sensitivity, 57.8%; specificity, 93.0%).

**Table 2:** Test Accuracies for the BeLPT

Test Modality	Sensitivity, % (95% CI)	Specificity, % (95% CI)	PPV, % (95% CI)	NPV, % (95% CI)
Sensitized vs nonsensitized				
Single test	64.5 (60.2-68.6)	93.9 (91.1-95.8)	92.7 (89.5-95.0)	68.6 (64.6-72.3)
Single test, high threshold	53.4 (49.3-57.3)	94.9 (92.6-96.5)	92.7 (89.5-95.0)	62.7 (59.2-66.1)
Single test, low threshold	70.6 (66.9-74.2)	77.5 (73.6-81.0)	79.1 (75.5-82.4)	68.6 (64.6-72.3)
Single PBMC test	64.8 (60.1-69.2)	93.4 (90.5-95.5)	91.5 (87.8-94.2)	70.8 (66.7-74.6)
Single BAL test	63.2 (51.9-73.1)	NA	NA	48.2 (35.4-61.2)
Split-sample test	79.9 (74.2-84.6)	85.1 (78.5-90.0)	89.1 (84.0-92.7)	73.7 (66.6-79.7)
CBD vs sarcoidosis				
Single test	61.5 (55.8-67.0)	90.8 (86.5-93.9)	88.9 (83.8-92.6)	66.5 (61.2-71.4)
Single test, high threshold	52.7 (47.3-58.0)	91.6 (87.6-94.4)	88.9 (83.8-92.6)	60.3 (55.4-65.0)
Single test, low threshold	67.1 (61.9-71.9)	83.2 (78.2-87.3)	83.6 (78.7-87.5)	66.5 (61.2-71.4)
Single PBMC test	61.2 (54.8-67.3)	90.0 (85.3-93.3)	86.3 (80.2-90.8)	69.2 (63.7-74.3)
Single BAL test	62.7 (50.0-73.9)	NA	NA	47.6 (33.4-62.3)
Split-sample test	76.0 (67.7-82.8)	80.4 (71.4-87.1)	82.9 (74.8-88.8)	72.9 (63.8-80.4)

Single test includes all individual BeLPTs performed (PBMCs and BAL, unless specified), and split-sample test includes the aggregated results of 2 simultaneous BeLPTs (either PBMCs or BAL). Unless otherwise specified, borderline results were excluded from the calculations. To account for the effect of borderline tests, test accuracies were calculated with borderline results counted as negative (high threshold) and positive (low threshold). Specificity and PPV were not calculated for BAL because by definition they would be 100%. BAL = bronchoalveolar lavage; BeLPT = beryllium lymphocyte proliferation test; CBD = chronic beryllium disease; NA = not applicable; NPV = negative predictive value; PBMC = peripheral blood mononuclear cell; PPV = positive predictive value.

Including borderline results using the high-threshold approach resulted in similar specificity of 94.9% (95% CI, 92.6-96.5) at substantially lower sensitivity of 53.4% (95% CI, 49.3-57.3). Using the low-threshold approach, a higher sensitivity of 70.6% (95% CI, 66.9-74.2) was achieved, but at the cost of a lower specificity of 77.5% (95% CI, 73.6-81.0).

The highest sensitivity of 79.9% (95% CI, 74.2-84.6) was achieved when considering split-sample tests as a unit, with moderate decline of specificity to 85.1% (95% CI, 78.5-90.0), which is mathematically explained by the reduction of true positive tests through merging.

A single positive test was associated with an LR of 10.5 (95% CI, 7.1-15.5) for true sensitization. A single negative test decreased the likelihood of sensitization, with an LR of 0.38 (95% CI, 0.33-0.43), whereas a single borderline did not influence the likelihood either way, with an LR of 0.99 (95% CI, 0.76-1.3). The presence of > 1 borderline test did not significantly increase the likelihood of true sensitization with an LR of 1.3 (95% CI, 0.73-2.3).

### CBD vs Sarcoidosis

The same calculations were repeated to differentiate only between patients with confirmed CBD and sarcoidosis. The sensitivity and specificity of a single test were 61.5% (95% CI, 55.8-67.0) and 90.8% (95% CI, 86.5-93.9), 52.7% (95% CI, 47.3-58.0) and 91.6% (95% CI, 87.6-94.4) for high threshold, 67.1% (95% CI, 61.9-71.9) and 83.2% (95% CI, 78.2-87.3) for low threshold, and 76.0% (95% CI, 67.7-82.8) and 80.4% (95% CI, 71.4-87.1) for split samples (Table 2). LRs were 6.3 (95% CI, 4.2-9.5) for positive tests, 1.7 (95% CI, 1.1-2.8) for borderline tests, and 0.40 (95% CI, 0.34-0.47) for negative tests.

### PBMC vs BAL BeLPT

**Figure 3:**
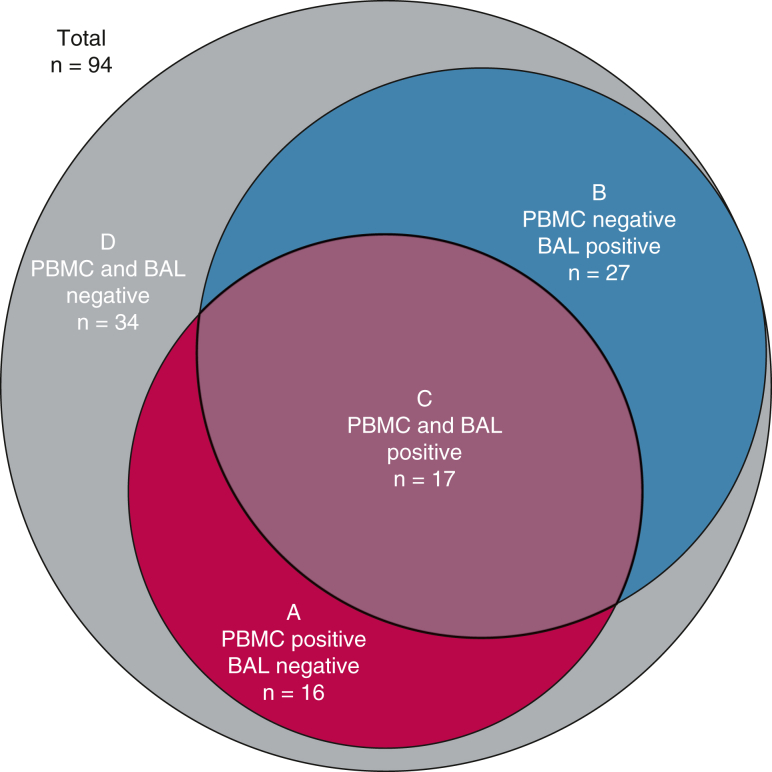
A-D, Proportional Venn diagram of PBMC and BAL BeLPT results. In total, both PBMC and BAL BeLPTs were performed in 94 patients, of which 60 (A, B, and C) met ATS criteria for sensitization (2 positive PBMC BeLPT or 1 positive and 1 borderline PBMC or 1 positive BAL BeLPT) and 34 (D) did not. In 33 cases, ATS criteria were fulfilled by PBMC BeLPTs (A and C). In 44 cases (B and C), ATS criteria were fulfilled by BAL BeLPT. Thus, ATS criteria were fulfilled only by PBMC BeLPT in 16 (A), only by BAL BeLPT in 27 (B), and independently by both PBMC and BAL BeLPT in 17 (C) cases. ATS = American Thoracic Society; BAL = bronchoalveolar lavage; BeLPT = beryllium lymphocyte proliferation test; PBMC = peripheral blood mononuclear cell.

There was no difference in sensitivity between PBMC and BAL BeLPTs (Table 2). Among 94 patients who underwent both PBMC and BAL BeLPTs, 60 were sensitized. In these patients, sensitization criteria were independently fulfilled by both PBMCs and BAL in 17 cases (28.3%), only by PBMCs in 16 cases (26.7%), and only by BAL in 27 cases (45.0%) (Fig 3).

### Clinical Characteristics

#### Pulmonary Function Tests

**Figure 4:**
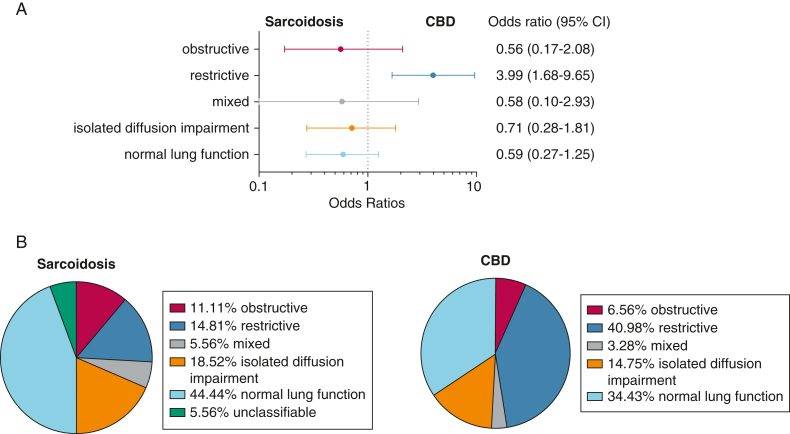
A, ORs of PFT patterns being attributed to sarcoidosis or CBD, presented as Forrest plots, and (B) proportional distribution of PFT patterns between sarcoidosis and CBD. The PFT pattern could not be classified in 3 patients with sarcoidosis because of missing diffusion capacity for carbon monoxide values. CBD = chronic beryllium disease; PFT = pulmonary function test.

Body plethysmography was available for 61 patients with CBD and 54 patients with sarcoidosis, and Dlco was available for 60 and 49 patients, respectively. Patients with CBD had significantly lower mean FVC, FEV_1_, total lung capacity, and Dlco. FEV_1_/FVC ratio did not differ between groups (Table 3). A restrictive pattern was significantly more common in CBD than in sarcoidosis. Normal lung function was numerically more frequent in sarcoidosis. Because of missing Dlco values, the phenotype could not be classified in 3 patients with sarcoidosis (e-Table 6, Fig 4, Table 3).

**Table 3:** Pulmonary Function Test Results of Patients With CBD and Sarcoidosis

Parameter	CBD	Sarcoidosis	*P* Value	Mean/Median Difference (95% CI)
FVC				
*z* score	−1.58 [1.47]	−0.75 [1.24]	.002^a^	0.82 (0.32 to 1.33)
% predicted	78.7 [19.8]	90.2 [16.4]		11.5 (4.7 to 18.2)
FEV_1_				
*z* score	−1.63 [1.44]	−0.93 [1.35]	.008^a^	0.70 (0.18 to 1.22)
% predicted	76.9 [20.8]	87.4 [18.5]		10.5 (3.2 to 17.8)
FEV_1_/FVC				
*z* score	−0.30 [1.14]	−0.36 [1.29]	.77	−0.07 (−0.52 to 0.38)
Ratio	0.77 [0.08]	0.77 [0.09]		0.00 (−0.03 to 0.003)
TLC				
*z* score	−1.49 [1.55]	−0.74 [1.27]	.006^a^	0.75 (0.22 to 1.28)
% predicted	82.5 [18.2]	91.5 [14.9]		9.0 (2.7 to 15.2)
Dlco % predicted				
*z* score	−1.26 (−2.67 to −0.27)	−0.90 (−1.90 to 0.24)	.04^a^	0.37 (0.03 to 1.30)
% predicted	78.4 [22.8]	88.6 [21.3]		10.1 (1.7 to 18.6)

Data are presented as mean [SD], median (interquartile range), or as otherwise indicated. CBD = chronic beryllium disease; Dlco = diffusion capacity for carbon monoxide; TLC = total lung capacity.

a Significant at α = .05.

#### Blood and BAL Markers

No differences were found in serum neopterin, soluble interleukin 2 receptor, angiotensin-converting enzyme, BAL differential cell counts, or CD4/CD8 (cluster of differentiation 4/cluster of differentiation 8) ratio between CBD and sarcoidosis (e-Tables 7, 8).

#### Extrapulmonary Findings

Extrapulmonary findings were reported in 24 patients (27.6%) with CBD and in 29 patients (27.4%) with sarcoidosis. A causal connection of extrapulmonary findings with CBD was considered conclusive in 15 patients (17.2%) and with sarcoidosis in 18 patients (17.0%). In the other cases, extrapulmonary manifestations were only suspected. The most common extrapulmonary findings in CBD were skin involvement (n = 10; 11.5%), arthralgias (n = 10; 11.5%), and eye involvement (n = 6; 6.9%). In sarcoidosis, these included skin involvement (n = 9; 8.5%), liver/spleen involvement (n = 9; 8.5%), and arthralgias (n = 7; 6.6%). We refrained from performing statistical comparisons between groups because of the lack of consistency in the reporting of extrapulmonary findings (e-Table 9).

## Discussion

Few studies have addressed the BeLPT in patients with manifest disease.^17^^,^^18^^,^^21^^,^^22^ To our knowledge, this is the largest analysis of the BeLPT in patients with granulomatous disease. A peak in proliferative response is observed early in the course of sensitization,^23^ possibly linked to a higher proportion of poorly proliferating beryllium-specific effector memory T cells in more advanced stages.^24^ In addition, proliferating T cells are detected in sarcoidosis,^25^ and nonspecific or bystander activation of T cells in cell culture might interfere with specific BeLPTs.^26^ Therefore, it seemed important to assess the performance of the BeLPT in this particular setting. We showed that the BeLPT can detect sensitization in manifest disease with high specificity. The main drawback of the BeLPT as a screening tool is its lack of sensitivity.^14^^,^^16^ We confirmed this finding in a diagnostic setting.

This finding may have been influenced by our testing strategy: negative BeLPTs were usually repeated until either positive results were obtained or CBD was considered ruled out. Once sensitization criteria were met, testing was usually discontinued. This led to accumulation of negative tests in some patients but not of positive tests, magnifying the effect of false-negatives on sensitivity. Also, to limit false-positives, we chose a conservative threshold of 3 SDs. Lowering the cutoff to 2 SDs increased sensitivity, but at the cost of unacceptably low specificity for a clinical setting. Results between 2 and 3 SDs neither increased nor decreased the likelihood of sensitization, which supports interpreting these results as borderline, which requires retesting. The lack of sensitivity can be countered by repeated testing, as shown by the merging of split-sample tests. In every fourth patient, ≥ 5 tests were required to prove sensitization (e-Table 5). On average, sensitized patients received 1.3 more tests than nonsensitized patients, which is explained by the need for confirmation after the first positive test.

Despite similar test accuracies, PBMC and BAL BeLPTs appeared complementary. When both were performed, 64.3% of sensitized patients had positive results in only 1 modality. This might be explained by the compartmentalization of beryllium-specific T cells.^27^ The addition of BAL proved beneficial because it identified sensitization in 27 cases where multiple PBMC BeLPTs had failed to demonstrate sensitization (45.0% of sensitized patients in whom BAL was performed). The high number of uninterpretable BAL BeLPTs should be noted. This was mostly because of low proliferation in positive control wells and can be attributed to inhibition of in vitro lymphocyte proliferation by alveolar macrophages^28^ and poor cell recovery in patients with obstructive ventilatory defect.

Assessing the accuracy of the BeLPT is limited by the lack of a reference standard other than the BeLPT itself. Therefore, true sensitization can only be confirmed by repeated BeLPT testing, as previously done by Stange et al.^14^ This self-confirmatory approach is inherently biased because the BeLPT serves as its own reference standard and, given the low sensitivity, likely underestimates the rate of sensitization. Some cases may have been misclassified as sarcoidosis simply because the BeLPT was not repeated often enough. Therefore, the proposed numbers can only be taken as approximations of the test accuracy.

We could not show differences in false-negative testing between patients with CBD receiving and not receiving immunosuppressive treatment. However, none received high-dose corticosteroids and only 5 received second-line treatment because we hold or taper immunosuppression before testing whenever possible, which we would generally advocate for (e-Table 10).

It has been demonstrated that cases of CBD could be detected when performing the BeLPT in metal-exposed patients formerly diagnosed with sarcoidosis^17^^,^^18^; however, this finding is inconsistently reported.^22^ In this study, the BeLPT revealed that 41.4% of metal-exposed patients who would otherwise have been misdiagnosed with sarcoidosis in fact had CBD. Only in 17 cases (8.1%) were we unable to make a clear distinction between CBD and sarcoidosis based on the BeLPT.

The BeLPT was performed in patients with suspected beryllium exposure, but because beryllium exposure is often occult, we broadly offer the BeLPT to patients with metal exposure. Except for electricians, who were underrepresented in CBD, occupations were similar across groups, with none of them emerging as more common in CBD than in sarcoidosis. Frequent occupations included those notoriously associated with high levels exposure (eg, machining) but also occupations with less conspicuous exposure, notably a high proportion of dental technicians, as previously observed,^17^^,^^29^ even though beryllium is no longer used in dental alloys in Germany.^17^^,^^30^ Thus, CBD should be considered as a differential diagnosis to sarcoidosis in a variety of work fields, not only in the beryllium industry itself, but also in downstream use of beryllium components, where awareness for beryllium exposure may not be given.^31^

Most patients in this analysis evidenced abnormal PFT results, which is striking when compared with other sarcoidosis cohorts.^32^^,^^33^ This might be related to their specific, potentially ongoing inhalational exposure. Patients with CBD exhibited even lower lung function and more restrictive patterns compared with sarcoidosis. Except for higher age in CBD, confounding factors (eg, smoking, comorbidities, obesity) did not differ between both groups. Therefore, we may postulate that CBD causes more severe lung function impairment than sarcoidosis. A recent study comparing CBD and beryllium-exposed sarcoidosis found lower lung function in sarcoidosis.^34^ However, patients with sarcoidosis were referred because of abnormal imaging, and patients with CBD were referred after positive screening on the BeLPT and generally had normal imaging. Conversely, in the present population, the cause for referral was almost universally disease manifestation, with the only difference between groups being the outcome of the BeLPT. Additionally, this demonstrates that in the absence of routine surveillance, BeS is identified late in its natural history. Earlier exposure cessation may have attenuated the clinical course of the patients.

Given the diversity and nonspecificity of extrapulmonary manifestations, and the difficulty in confirming most of them, an etiologic connection to the disease often remained presumptive. As such, we cannot accurately deduce the true prevalence of extrapulmonary manifestations in this cohort, especially because they were not evaluated systematically. Nonetheless, we think it is noteworthy that several patients with CBD displayed nonrespiratory symptoms, with a similar proportion as those with sarcoidosis. These mainly included eye and skin manifestations, well-recognized manifestations of CBD,^7^ but on some occasions we also found involvement of internal organs (e-Table 9).

The study is limited by its retrospective design, which might imply inconsistencies in data acquisition. Because our center performs the BeLPT for external referrals, we had access to blood samples but lacked clinical and lung functional data in some cases. Similarly, follow-up data were too scarce to make longitudinal assumptions. The included patients may not be representative of the general sarcoidosis population because they share specific exposures which occasioned the BeLPT, whereas other patients with sarcoidosis likely have various exposure backgrounds. Given these selection biases, we cannot infer the true prevalence of CBD in larger populations of patients with apparent sarcoidosis.

## Interpretation

The BeLPT is highly specific for detecting CBD in patients with granulomatous lung disease. However, its poor sensitivity must be considered. Importantly, a single negative BeLPT is not sufficient to rule out CBD. Repeated testing and the addition of BAL may be necessary to make a reliable diagnosis. Despite the low sensitivity of the BeLPT, we were often able to diagnose CBD in patients who would otherwise have been misdiagnosed with sarcoidosis. This underscores the importance of considering CBD as a differential diagnosis to sarcoidosis in patients with a history of metal exposure. Accurately identifying patients with CBD is crucial not only for eliminating the source of exposure, but also because this finding may indicate a more unfavorable course of disease and notably, more severely impaired lung function.

## Funding/Support

B. C. F. was supported by a research grant from the Berta-Ottenstein-Program for Advanced Clinical Scientists from the 10.13039/501100021729Faculty of Medicine, University of Freiburg, and J. M.-Q. was supported by research grants from Advita Lifescience and the 10.13039/501100001659German Research Foundation (MU 692/2-1).

## Financial/Nonfinancial Disclosures

The authors have reported to *CHEST* the following: L. J. reports lecture fees, and congress and travel support from Boehringer Ingelheim. G. Z. reports congress and travel support from the Swiss Respiratory Society (SGP). P. A. reports lecture fees from Boehringer Ingelheim. D. S. reports lecture fees from Boehringer Ingelheim, and congress and travel support from Chiesi and CSL Behring. J. M.-Q. reports research grants from Advita Lifescience, the German Research Foundation, and Bristol Myers Squibb; consulting fees from Advita Lifescience; lecture fees from AstraZeneca, CSL Behring, Actelion, and Roche; congress and travel support from Boehringer Ingelheim and CSL Behring; advisory board participation for Boehringer Ingelheim; and holds patents (WO2020225246A1, WO2021152119A1, and related). D. S. reports honoraria from AstraZeneca, Berlin-Chemie/Menarini, Boehringer Ingelheim, Chiesi, CSL Behring, Curetis AG, GSK, Merck, MSD, Novartis, Sanofi, Vifor, and Roche; board participations for AstraZeneca, Berlin-Chemie/Menarini, Boehringer Ingelheim, Chiesi, CSL Behring, Curetis AG, GSK, Merck, MSD, Roche, Novartis, Sanofi, and Vifor; and is a Global Initiative for Chronic Obstructive Lung Disease (GOLD) representative for Switzerland. B. C. F. reports research grants from Bristol Myers Squibb and Relief Therapeutics; consulting fees from Advita Lifescience; lecture fees from AstraZeneca, Boehringer Ingelheim, Vifor, Roche, Novartis, and Actelion; congress and travel support from Boehringer Ingelheim; advisory board participation for Boehringer Ingelheim; and holds patents (WO2020225246A1, WO2021152119A1, and related). None declared (C. T., E. W., C. Q.).

## Supplementary Data

e-Online Data
