# Supplementary material for: Beryllium Lymphocyte Proliferation Test: Differential Diagnosis of Sarcoidosis and Chronic Beryllium Disease
Source: Chest. 2025 Jul 1;168(6):1404–14. doi: 10.1016/j.chest.2025.06.034 (PMC12831088; doi:10.1016/j.chest.2025.06.034)
Supplement: e-Online Data [file mmc1.docx]

Supplementary material to *Beryllium Lymphocyte Proliferation Test: Differential Diagnosis of Sarcoidosis and Chronic Beryllium Disease*

e-Appendix 1: Description of sarcoidosis and CBD diagnostic algorithm

In patients with suspected sarcoidosis, our basic workup starts with clinical examination and establishing patients’ history (symptoms, family history, comorbidities, medication, travel, exposures and occupation). We then perform bodyplethysmography and measurement of diffusion capacity for carbon monoxide, as well as laboratory tests. These include differential blood count, electrolytes, creatinine, transaminases, soluble interleukin-2 receptor, neopterine, interferon-gamma-release assay or tuberculin skin test for tuberculosis, HIV serology, immunoglobulin levels, antinuclear antibodies, antineutrophil cytoplasmatic antibodies, rheumatoid factor, anti-citrullinated protein antibodies in all patients, and in presentations suggestive of hypersensitivity pneumonitis, specific immunoglobulins G.

For baseline imaging and biopsy planning, we nowadays perform high-resolution computed tomography in almost all cases. While the diagnosis formerly mainly relied on forceps biopsies and surgical techniques like mediastinoscopy or surgical lung biopsies, we nowadays primarily perform endobronchial ultrasound-guided needle biopsies. During bronchoscopy, bronchoalveolar lavage is performed to exclude infection by means of bacterial, mycobacterial and fungal cultures and polymerase chain reaction, as well as for differential cell count. In cases with mainly extrapulmonary involvement, organ biopsies may be performed as indicated.

When occupational history indicates possible beryllium exposure in patients presenting with sarcoidosis-like disease, we offer BeLPT. A first set of two blood BeLPTs is generally performed concurrently to or after the aforementioned general workup. In cases of negative or inconclusive results and high clinical suspicion of CBD we offer repeated testing, generally involving both blood and BAL BeLPT. Besides our own patients that are tested at time of diagnosis, we get outside referrals for re-evaluation of formerly established diagnoses of “sarcoidosis”.

We almost never test asymptomatic individuals, except healthy subjects for quality control who were not included in the present study. This is due to the fact that routine occupational screening is neither required nor reimbursed in Germany. Therefore, virtually all patients present with an initial suspicion of sarcoidosis based on symptoms or incidental imaging findings.

All beryllium lymphocyte proliferation tests were performed at the same laboratory following the same protocol. After isolation of mononuclear cells by density gradient centrifugation from blood or BAL samples, cells were cultured in 96-well culture plates at a density of 5 x 10^5^ cells/ml in 100 µl culture medium per well (RPMI 1640 (Gibco Life Technologies, Darmstadt, Germany)) containing 10% NU medium (Fisher Scientific GmbH, Schwerte, Germany) and 1% penicillin/streptomycin (Biochrom, Berlin, Germany). In 32 wells, cells were cultured without stimulation as negative controls and to quantify background proliferation. Four wells each with two mitogens (phytohaemagglutinin (PHA; 5 µg/ml); concanavalin A (ConA; 10 µg/ml)) served as positive controls. In the remaining wells, cells were cultured with beryllium sulphate (BeSO4) in 6 concentrations ranging from 100 pM to 10 µM, 8 wells each. On days 3 and 6, 5-bromo-2'-deoxyuridine (BrdU) and 2'-deoxycytidine were added at a concentration of 2.5 mM. On days 4 and 7, the plates were washed 5 times with phosphate-buffered saline, fixed in ice-cold 70% ethanol and air-dried. BrdU incorporation was measured by enzyme-linked immunosorbent assay (ELISA) using a horseradish peroxidase-conjugated anti-BrdU antibody (Roche Diagnostics, Mannheim, Germany) and 3,3′,5,5′-tetramethylbenzidine (TMB) as chromogen, measured using a standard ELISA reader (PerkinElmer, Rodgau, Germany) and reported as optical density (OD).

After data clearance (removal of negative values and outliers), A BeLPT was considered uninterpretable in the following cases: poor proliferative response in both mitogen controls (i.e. not reaching the positivity threshold); high statistical variance in OD (coefficient of variation (CV) > 0.3 in test or control rows), or more than 50% negative wells per concentration (cell death). Stimulation indices (SI) were calculated for each BeSO_4_ concentration and mitogen controls by dividing their background-subtracted mean OD by the background-subtracted mean OD in the unstimulated wells. We calculated individual positivity thresholds based on the unstimulated wells rather than a fixed SI to account for variability in background proliferation,^25^ as our patients suffer from diseases with variable amounts of proliferating cells. The positivity threshold was defined as the mean OD in the unstimulated wells + three standard deviations (SD). A BeLPT was considered positive if at least two BeSO4 concentrations on day 4 or 7 had SI above *control + 3 SD.* It was considered negative if all SI on days 4 and 7 lay below *control + 2 SD*. The others (≥ 1 SI above *control + 2 SD*, but ≤ 1 SI above *control + 3 SD*) were considered borderline.

e-Appendix 2: Description of BeLPT procedure

e-Table 1 Overview of LPT Characteristics

|  | absolute number (%) |
| --- | --- |
| **Overall LPTs performed** | **1317** |
| Excluded LPTs^a^ | 83 (6.3) |
| **Included LPTs** | **1234 (93.7)** |
| **Of which PBMC** | **1084 (87.8)** |
| Positive | 324 (29.9) |
| Borderline | 182 (16.8) |
| Negative | 553 (51.0) |
| Uninterpretable | 25 (2.3) |
| **Of which BAL** | **150 (12.2)** |
| Positive | 51 (34.0) |
| Borderline | 20 (13.3) |
| Negative | 60 (40.0) |
| Uninterpretable | 19 (12.7) |
| ^a^ Test result unavailable  LPT = lymphocyte proliferation test, PBMC = peripheral blood mononuclear cells, BAL = bronchoalveolar lavage | |

e-Table 2 Sites and modalities of histological proof of granulomas in patients with CBD and sarcoidosis

| Site/modality of biopsy | CBD (n (%)) | sarcoidosis (n (%)) |
| --- | --- | --- |
| Transbronchial forceps biopsy | 23 (26.1) | 19 (17.9) |
| Transbronchial needle aspiration of lymph nodes | 21 (23.9) | 11 (10.4) |
| Mediastinoscopy | 8 (9.1) | 12 (11.3) |
| Surgical lung biopsy | 6 (6.8) | 10 (9.4) |
| Endobronchial biopsy | 5 (5.7) | 9 (8.5) |
| Transbronchial cryobiopsy | 4 (4.5) | 2 (1.9) |
| Transthoracic needle biopsy | 1 (1.1) | 0 (0) |
| Extrapulmonary biopsy (e.g. skin) | 6 (6.8) | 12 (11.3) |
| Unknown biopsy site^a^ | 20 (22.7) | 16 (15.1) |
| No biopsy/unknown whether biopsy was performed | 4 (4.5)^b^ | 21 (19.8)^c^ |
| CBD = chronic beryllium disease  Percentages refer to the total number of patients. Some patients had more than one granuloma-positive biopsy site  ^a^ Biopsies performed at referring hospitals, original histology reports unavailable.  ^b^ 4 patients in whom diagnosis was made upon typical clinical and radiological manifestation with proven exposure and sensitisation.  ^b^ 7 patients with typical clinical and radiological manifestation, 3 patients with Löfgrens’s syndrome, and 11 patients who were diagnosed elsewhere, for whom it was not evident from our records whether histological proof was obtained. | | |

e-Table 3 Occupational history of patients with CBD and sarcoidosis

| Occupation | CBD (n (%)) | sarcoidosis (n (%)) | p | OR (95% CI) |
| --- | --- | --- | --- | --- |
| Metal worker (unspecified) | 15 (15.6) | 14 (13.2) | 0.69 | 1.22 (0.58-2.59) |
| Dental technician | 14 (14.6) | 11 (10.4) | 0.40 | 1.48 (0.64-3.27) |
| Machinist | 10 (10.4) | 12 (11.3) | >0.99 | 0.91 (0.37-2.09) |
| Welder/solderer | 8 (8.3) | 9 (8.5) | >0.99 | 0.98 (0.36-2.49) |
| Mechanic (unspecified) | 9 (9.4) | 5 (4.7) | 0.27 | 2.09 (0.69-5.72) |
| Electrician | 2 (2.1) | 10 (9.4) | 0.04* | 0.20 (0.04-0.93) |
| Toolmaker | 7 (7.3) | 5 (4.7) | 0.55 | 1.59 (0.53-4.54) |
| Caster | 7 (7.3) | 3 (2.8) | 0.20 | 2.70 (0.72-9.79) |
| Aeronautical industry worker | 3 (3.1) | 2 (1.9) | 0.67 | 1.68 (0.34-9.59) |
| Automobile industry worker/car mechanic | 1 (1.0) | 4 (3.8) | 0.37 | 0.27 (0.02-1.67) |
| Precision technician | 2 (2.1) | 0 | 0.22 | +∞ |
| Environmental exposure | 2 (2.1) | 0 | 0.22 | +∞ |
| Other | 16 (16.7) | 31 (29.2) | 0.05* | 0.48 (0.25-0.96) |
| Total^†^ | 96 (100) | 106 (100) |  |  |
| CBD = chronic beryllium disease, CI = confidence interval, OR = odds ratio  * significant  ^†^ total of reported occupations. Some patients reported more than one occupation.  OR > 1 favoured CBD over sarcoidosis  Odds ratios for *precision technicians* and *environmental exposure* were calculated as +∞, since they did not occur in the sarcoidosis group. | | | | |

e-Table 4 number of negative and borderline tests up to the first positive test in sensitised patients

|  | number of negative or borderline tests (%) | | | | | | | average |
| --- | --- | --- | --- | --- | --- | --- | --- | --- |
|  | 0 | 1 | 2 | 3 | 4 | 5 | 6 |  |
| CBD |  |  |  |  |  |  |  |  |
| Negative | 52 (59.7) | 21 (24.1) | 11 (12.6) | 1 (1.1) | 1 (1.1) | 1 (1.1) | 0 | 0.6 |
| Borderline | 64 (73.6) | 19 (21.8) | 2 (2.3) | 2 (2.3) | 0 | 0 | 0 | 0.3 |
| Negative or borderline | 39 (44.8) | 25 (28.7) | 15 (17.2) | 5 (5.7) | 2 (2.3) | 0 | 1 (1.1) | 1.0 |
| All sensitised |  |  |  |  |  |  |  |  |
| Negative | 112 (65.9) | 36 (21.2) | 19 (11.2) | 1 (0.6) | 1 (0.6) | 1 (0.6) | 0 | 0.5 |
| Borderline | 115 (67.6) | 47 (27.6) | 4 (2.3) | 4 (2.3) | 0 | 0 | 0 | 0.4 |
| Negative or borderline | 76 (44.7) | 57 (33.5) | 23 (13.5) | 9 (5.3) | 3 (1.7) | 1 (0.6) | 1 (0.6) | 0.9 |
| CBD = chronic beryllium disease  The numbers represent the patients with the according amount of negative or borderline tests performed prior to or on the same day as the first positive test. | | | | | | | |  |

e-Table 5 total and average number of tests performed

|  | total number of tests (%) | | | | | | | | average |
| --- | --- | --- | --- | --- | --- | --- | --- | --- | --- |
|  | 1 | 2 | 3 | 4 | 5 | 6 | 7 | 8 |  |
| CBD | 0 | 19 (21.8) | 18 (20.7) | 24 (27.8) | 10 (11.5) | 8 (9.2) | 4 (4.6) | 4 (4.6) | 3.98 |
| Sarcoidosis | 18 (17.0) | 53 (50.0) | 11 (10.4) | 15 (14.2) | 4 (3.8) | 2 (1.9) | 2 (1.9) | 1 (0.9) | 2.56 |
| All sensitised | 3 (1.8) | 56 (32.9) | 28 (16.5) | 41 (24.1) | 15 (8.8) | 16 (9.4) | 5 (2.9) | 6 (3.5) | 3.63 |
| All non-sensitised | 39 (17.6) | 128 (57.9) | 19 (8.5) | 26 (11.8) | 4 (1.8) | 2 (0.9) | 2 (0.9) | 1 (0.5) | 2.31 |
| CBD = chronic beryllium disease | | | | | | | | | |

e-Table 6 frequency of lung function patterns in patients with CBD and sarcoidosis

| Pattern | CBD (n (%)) | sarcoidosis (n (%)) | p | OR (95% CI) |
| --- | --- | --- | --- | --- |
| Obstructive | 4 (6.6) | 6 (11.1) | 0.51 | 0.56 (0.17-2.08) |
| Restrictive | 25 (41.0) | 8 (14.8) | 0.002 | 3.99 (1.68-9.65) |
| Mixed | 2 (3.3) | 3 (5.6) | 0.66 | 0.58 (0.10-2.93) |
| Isolated diffusion impairment | 9 (14.8) | 10 (19.6) | 0.61 | 0.71 (0.28-1.81) |
| Normal | 21 (34.4) | 24 (47.1) | 0.18 | 0.59 (0.27-1.25) |
| CBD = chronic beryllium disease, CI = confidence interval, OR = odds ratio  Percentages and odds ratios are calculated based on the available data for each pattern. Because of missing diffusion capacity measurements, 3 patients with sarcoidosis who had normal lung volumes could not be classified within *isolated diffusion impairment* or *normal*. Thus, for sarcoidosis, ventilatory patterns are calculated based on a total of n = 54, *isolated diffusion impairment* and *normal* on a total of n = 51. Note: in figure 4b of the main article, all percentages are calculated based on n = 54, which leaves 5.6% unclassifiable patients, who might have either isolated diffusion impairment or normal lung function. | | | | |

e-Table 7 BAL differential cell counts in patients with CBD and sarcoidosis

| BAL composition | CBD (mean (SD)) | sarcoidosis (mean (SD)) | p |
| --- | --- | --- | --- |
| Absolute cell count (10^6^) | 25.0 (18.9) | 25.6 (20.8) | 0.572 |
| Cells/100 ml (10^6^) | 14.4 (9.1) | 14.0 (10.2) | 0.267 |
| Macrophages (%) | 57.1 (22.1) | 64.3 (17.3) | 0.076 |
| Lymphocytes (%) | 34.6 (22.3) | 32.0 (16.8) | 0.510 |
| Neutrophils (%) | 6.5 (13.5) | 2.6 (4.5) | 0.050 |
| Eosinophils (%) | 1.7 (3.0) | 1.1 (1.9) | 0.086 |
| CD4/CD8 ratio | 4.2 (4.0) | 4.5 (3.9) | 0.305 |
| Number of BAL procedures (n) | 49 | 38 |  |
| BAL = bronchoalveolar lavage, CBD = chronic beryllium disease, CD = cluster of differentiation, CI = confidence interval, OR = odds ratio, SD = standard deviation | | | |

e-Table 8 Baseline serum markers in patients with CBD and sarcoidosis

| Serum markers | CBD | sarcoidosis | p |
| --- | --- | --- | --- |
| ACE level available, n | 31 | 57 |  |
| ACE (U/l), mean (SD) | 59.8 (59.5) | 60.8 (49.1) | 0.684 |
| Elevated ACE, n (%) | 7 (22.6) | 10 (17.5) | 0.582 |
| sIL2R level available, n | 48 | 65 |  |
| sIL2R (U/ml), mean (SD) | 873 (836) | 964 (643) | 0.279 |
| Elevated sIL2R, n (%) | 30 (62.5) | 41 (63.1) | >0.999 |
| Neopterin level available, n | 50 | 62 |  |
| Neopterin (nmol/l), mean (SD) | 12.9 (7.0) | 13.3 (13.0) | 0.602 |
| Elevated neopterin, n (%) | 26 (52.0) | 34 (54.8) | 0.850 |
| ACE = angiotensin converting enzyme, CBD = chronic beryllium disease, OR = odds ratio, sIL2R = soluble interleukin 2 receptor, SD = standard deviation  Reference values: ACE 12-82 U/l, sIL2R 158-623 U/ml, neopterin < 10 nmol/l | | | |

e-Table 9 extrapulmonary findings of patients with CBD and sarcoidosis

|  | CBD (n (%)) | | | sarcoidosis (n (%)) | | |
| --- | --- | --- | --- | --- | --- | --- |
|  | established | suspected | total | established | suspected | total |
| Eye | 3 (4.1) | 3 (4.1) | 6 (8.1) | 4 (4.8) | 0 (0) | 4 (4.8) |
| Skin | 9 (12.2) | 1 (1.4) | 10 (13.5) | 8 (9.8) | 1 (1.2) | 9 (10.9) |
| Joint | 2 (2.7) | 8 (10.8) | 10 (13.5) | 3 (3.7) | 4 (4.8) | 7 (8.5) |
| Heart | 1 (1.4) | 1 (1.4) | 2 (2.7) | 0 (0) | 2 (2.4) | 2 (2.4) |
| Kidney | 1 (1.4) | 0 (0) | 1 (1.4) | 0 (0) | 3 (3.7) | 3 (3.7) |
| Central nervous system | 1 (1.4) | 0 (0) | 1 (1.4) | 1 (1.2) | 1 (1.2) | 2 (2.4) |
| Muscle | 1 (1.4) | 0 (0) | 1 (1.4) | 0 (0) | 0 (0) | 0 (0) |
| Liver/spleen | 3 (4.1) | 0 (0) | 3 (4.1) | 7 (8.5) | 2 (2.4) | 9 (10.9) |
| Gastrointestinal system | 1 (1.4) | 0 (0) | 1 (1.4) | 2 (2.4) | 0 (0) | 2 (2.4) |
| Salivary glands | 2 (2.7) | 0 (0) | 2 (2.7) | 0 (0) | 0 (0) | 0 (0) |
| Other* | 2 (2.7) | 0 (0) | 2 (2.7) | 3 (3.7) | 1 (1.2) | 4 (4.8) |
| Total data available | 74 (100) | | | 82 (100) | | |
| CBD = chronic beryllium disease  *other involved organs. In CBD: nose (n = 1), bone (n = 1). In sarcoidosis: bone marrow (n = 1), axillary lymph node (n = 1), bone (suspected, n = 1). | | | | | | |

e-Table 10 Medication at time of testing

|  | Testing timepoints (n (%)) |
| --- | --- |
| No treatment | 336 (87.0) |
| Low-dose corticosteroids^a^ | 30 (7.8) |
| High-dose corticosteroids^b^ | 4 (1.0) |
| Corticosteroids (dose unknown) | 7 (1.8) |
| Azathioprine + low-dose corticosteroids^a^ | 5 (1.3) |
| Methotrexate + low-dose corticosteroids^a^ | 2 (0.5) |
| Adalimumab | 2 (0.5) |
| Total available | 386 (100) |
| Treatment status unkown^c^ | 346 |
| ^a^ ≤ 7.5 mg prednisone  ^b^ 10 mg prednisone in 3 cases, 15 mg prednisone in one case. (1 positive, 3 negative test results)  ^c^ Mostly patients referred only for BeLPT-testing, patient charts unavailable. Referring physicians are advised to hold or taper immunosuppressive treatment to the lowest achievable dose before BeLPT. | |
